# Primary Carcinoma of Appendix

**Published:** 1915-11

**Authors:** 


**Primary Carcinoma of Appendix.** J. W. Markoe, Bull. of Lying-in Hospital of City of N. Y., Sept. MacCarty and McGrath found 1 in every 225 appendixes operated on, and 1 in every 53 partially or wholly obliterated, to be cancerous. In a subsequent series of 3039 specimens, 18 (0.6%) were cancerous, the lesion occurring at or near the tip of all. The author's case occurred in a married woman, aged 34, who had had one miscarriage and 3 children. The pathologic diagnosis was chronic appendicitis with spheroid cell carcinoma displacing the mucosa of the obliterated portion. There was no distinct tumor. There was also found a serous cyst of the broad ligament, of the size of an orange.

